# Estimating the Post-Mortem Interval Under Extreme Heat Environments: A Climate-Adaptive Case Series Based on Artificial Intelligence-Supported Diagnostics

**DOI:** 10.3390/diagnostics16091407

**Published:** 2026-05-06

**Authors:** Francesco Sessa, Clelia Grippaldi, Massimiliano Esposito, Carlos A. Gutierrez, Emina Dervišević, Efehan Ulas, Federica Ministeri, Lucio Di Mauro, Matteo Bolcato, Cristoforo Pomara, Monica Salerno

**Affiliations:** 1 Department of Psychology and Health Sciences, Faculty of Human Sciences, Education, and Sports, Pegaso University, 80143 Naples, Italy; 2 Department of Medical, Surgical and Advanced Technologies “G.F. Ingrassia”, University of Catania, 95121 Catania, Italy; grpclg02b63c351z@studium.unict.it (C.G.); federicaministeri@gmail.com (F.M.); dr.luciodimauro@gmail.com (L.D.M.); cristoforo.pomara@unict.it (C.P.); monica.salerno@unict.it (M.S.); 3 Faculty of Medicine and Surgery, “Kore” University of Enna, 94100 Enna, Italy; massimiliano.esposito@unikore.it; 4 Forensic Sciences Unit, School of Natural Sciences and Mathematics, Chaminade University of Honolulu, Honolulu, HI 96816, USA; carlos.gutierrez@chaminade.edu; 5 Department of Forensic Medicine, Faculty of Medicine, University of Sarajevo, 71000 Sarajevo, Bosnia and Herzegovina; emina.dervisevic@mf.unsa.ba; 6 Faculty of Medicine, Department of Biostatistics and Medical Informatics, Kirklareli University, Kirklareli 39060, Türkiye; efehanulas@klu.edu.tr; 7 Department of Medicine, Saint Camillus International University of Health and Medical Sciences, 00131 Rome, Italy; matteo.bolcato@unicamillus.org

**Keywords:** Post-Mortem Interval (PMI), forensic diagnostics, forensic pathology, climate-driven decomposition, hyperthermal environments, AI-Assisted PMI Estimation, machine learning, decomposition dynamics, mediterranean climate

## Abstract

**Background/Objectives**: Accurate post-mortem interval (PMI) estimation becomes increasingly difficult when bodies decompose under extreme heat. Hyperthermal Mediterranean environments accelerate soft-tissue degradation, induce early mummification, and distort classical thanatological indicators, often resulting in substantial PMI overestimation. This study analyzes three forensic cases affected by climate-driven decomposition anomalies and presents a climate-adaptive, AI-assisted diagnostic framework applied uniformly across all cases to improve PMI interpretation. **Methods:** A retrospective case series analysis was conducted on three individuals recovered during summer heatwaves. Crime scene investigation, post-mortem computed tomography (PMCT), autopsy, and genetic identification were integrated with 5–15-year meteorological datasets. Classical PMI estimations were compared with circumstantial data. A multimodal AI model, incorporating environmental features, decomposition morphology, and microenvironmental modifiers, was operationalized for each case using a hybrid Random Forest–LSTM architecture. Engineered indices included Accumulated Degree Days (ADD), a Decomposition Index, and climate-stress metrics (Thermal Load Index, Desiccation Pressure Factor, Microenvironmental Distortion Coefficient). Quantile regression provided calibrated prediction intervals. **Results:** Morphological assessments overestimated PMI in every case, suggesting intervals of 1–6 months despite true PMIs of approximately 20 days (Cases 1–2) or 36–48 h (Case 3). The AI model yielded conceptual outputs more consistent with verified PMIs, ~21 days (Case 1), ~23 days (Case 2), and ~42 h (Case 3), each accompanied by 50% and 90% prediction intervals. Explainability analyses identified thermal load, desiccation pressure, and microenvironmental distortion, particularly insulation in Case 3, as dominant drivers. **Conclusions:** Extreme heat fundamentally alters decomposition trajectories, rendering classical PMI methods unreliable. Applying a climate-aware, AI-assisted diagnostic framework across all three cases improved interpretability, providing uncertainty-aware estimates aligned with true PMIs. The AI framework is presented as a conceptual, non-trained, proof-of-concept system, and reported outputs represent operational demonstrations rather than validated predictions, offering a promising foundation for next-generation PMI diagnostics in hyperthermal forensic settings.

## 1. Introduction

Estimating the post-mortem interval (PMI) remains one of the central challenges in forensic pathology, as it underpins the reconstruction of peri-mortem events and guides medico-legal decision-making. Classical frameworks (algor, livor, and rigor mortis, as well as later-stage decomposition markers) have historically provided the foundation for PMI estimation, yet their applicability decreases significantly with advancing decomposition or when environmental variables deviate from expected patterns. Recent systematic analyses confirm that traditional signs retain acceptable reliability primarily within the first 2–3 post-mortem days, after which accuracy declines sharply as autolysis, putrefaction, insect activity, and tissue loss introduce substantial biological variability [1,2,3,4,5,6,7,8,9,10,11].

Over the past decade, climate change has emerged as a transformative factor in forensic taphonomy. Mediterranean and tropical regions increasingly experience prolonged heatwaves, elevated baseline summer temperatures, and altered humidity cycles, conditions shown to accelerate microbial and enzymatic activity, promote rapid desiccation, and modify insect colonization patterns [12,13]. These environmental shifts generate non-linear decomposition trajectories that diverge markedly from the assumptions embedded in reference models such as Henssge’s nomogram or TBS (total body score)/ADD (Accumulated Degree Days) curves, thereby contributing to systematic PMI misestimation in real-world cases [14,15,16,17,18,19,20].

Concurrently, forensic science is undergoing a technological shift driven by artificial intelligence. Machine learning approaches, including Random Forests, neural networks, and multimodal architectures, have demonstrated substantial promise in PMI prediction when applied to microbial, molecular, and environmental datasets. Systematic reviews show that AI-based models outperform traditional methods under diverse environmental conditions, achieving markedly reduced prediction errors and improved robustness across tissues and substrates [21,22,23]. Beyond PMI, AI is increasingly integrated into forensic pathology more broadly, supporting post-mortem imaging, injury interpretation, and biological pattern analysis, while simultaneously raising the need for transparent and interpretable diagnostic tools [24].

Within this evolving landscape, there is a growing demand for climate-adaptive, explainable, and operationally feasible diagnostic models capable of integrating environmental data, decomposition features, and microenvironmental modifiers. Such tools are essential for enhancing PMI accuracy in hyperthermal settings, where classical markers routinely fail. The present study responds to this need by examining three Mediterranean forensic cases characterized by extreme environmental exposure and decomposition time mismatch, and by outlining an AI-assisted diagnostic framework designed to generate climate-aware PMI estimates supported by transparent uncertainty reporting. Accordingly, this study does not aim to validate an AI model or report predictive performance. Instead, it introduces and operationalizes a conceptual, climate-adaptive AI framework to demonstrate how multimodal data integration may reinterpret PMI under hyperthermal conditions.

## 2. Materials and Methods

### 2.1. Case Selection

This study was designed as a retrospective forensic case series aimed at illustrating the conceptual operationalization of a climate-adaptive, AI-assisted framework for post-mortem interval (PMI) estimation under hyperthermal conditions. Three medico-legal autopsies performed between 2018 and 2025 at the University of Catania were reviewed. From an archive of ~350 cases, the following predefined inclusion criteria were applied:

• Recovery during the summer season;
• Presence of advanced decomposition (mummification, extensive tissue loss, or partial skeletonization);
• Marked discrepancy between morphological indicators and circumstantial PMI.

These three cases were intentionally selected as extreme climate-distortion scenarios to demonstrate the interpretive behavior of the conceptual AI model rather than to evaluate predictive performance.

The study protocol was reviewed and approved by the Ethics Committee of Hospital ‘Rodolico’ of Catania (approval number 04/CEL; date: 15 January 2025).

### 2.2. Crime Scene Investigation (CSI)

Each scene examination followed institutional forensic standards, including wide, mid-range, and close-up photographic documentation; mapping of the body’s position; and characterization of environmental and microclimatic factors (sun exposure, shading, terrain type, vegetation, and ambient conditions). Where available, ambient temperature, humidity, and wind data were recorded. Presence of fauna, insects, mud, vegetative debris, and coverings (e.g., isothermal blanket) was documented.

### 2.3. Post-Mortem Computed Tomography (PMCT)

Whole-body PMCT was conducted prior to autopsy using the institutional forensic imaging protocol. Examinations assessed skeletal integrity, distribution of decomposition gases, preservation of soft tissues, internal anatomical structures, and evidence of trauma. In cases with advanced putrefaction or skeletonization, PMCT served mainly to document residual gas patterns, structural collapse, and regions of anatomical loss.

### 2.4. Autopsy Examination

Autopsies were performed in accordance with national medicolegal standards. External inspection documented decomposition stage (mummification, maceration, parchment-like desiccation, skeletonization), insect damage, and remaining soft-tissue structures. Internal examination was performed when anatomic preservation allowed, focusing on organ remnants, putrefactive gas distribution, and evidence of injury. In cases with complete organ destruction, cause-of-death assessment was limited.

### 2.5. Histological Analysis

When preserved tissue fragments were available (heart, lung, brain, skin), they were processed with standard histological techniques (FFPE preparation, microtomy, and routine staining). Microscopic evaluation assessed tissue architecture, connective-tissue preservation, autolytic changes, and the degree of putrefactive degradation. In some cases, histology was not feasible due to complete tissue destruction.

### 2.6. Genetic Identification

DNA extraction was performed on any available preserved soft tissues (e.g., plantar fascia, temporal or dorsal soft tissues, residual organs). Standard automated forensic extraction kits and multiplex STR systems were used, followed by capillary electrophoresis. STR profiles were compared with known relatives when required. In Case 3, identification was achieved through hospital bracelet documentation.

### 2.7. Meteorological Data Collection

For each disappearance-to-discovery interval, daily meteorological data were retrieved from a regional weather station, including minimum/maximum/mean temperature, humidity, atmospheric pressure, wind velocity, and visibility. These measurements were compared with historical datasets covering 5–15 previous years to identify seasonal anomalies, which are central drivers of climate-accelerated decomposition. Meteorological gaps were imputed using station short-gap means.

### 2.8. Classical PMI Estimation

Classical PMI estimations were derived from morphology-based decomposition timelines and, where applicable, entomological indicators, with reference to the established forensic literature addressing post-mortem changes under varying environmental conditions [6,7,10,14]. Due to advanced decomposition, rectal temperature, body mass, and key thermometric variables were unavailable, limiting the applicability of Henssge’s nomogram and similar models. The results were interpreted cautiously in light of advanced decomposition and extreme heat exposure, circumstances known to accelerate tissue degradation and distort conventional timelines. Finally, PMI estimations were compared with verified circumstantial PMIs.

### 2.9. AI-Assisted PMI Model: Application to All Cases

The climate-adaptive AI framework previously described in the Supplementary Material was applied retrospectively to all three cases (Supplementary Files S1 and S2). This model integrates environmental, morphological, and microenvironmental data using a multimodal architecture to produce PMI estimates with uncertainty ranges. The AI system applied in this study is a conceptual, non-trained framework, not a validated predictive model.

#### 2.9.1. Input Data

For each case, the following categories of predictors were extracted:

• Environmental variables:

Daily temperature (min/mean/max), humidity, pressure, wind velocity, visibility, and historical climate comparisons.

• Morphological variables:

Degree of mummification or desiccation, soft-tissue loss, skeletal exposure, presence of rigor, insect activity, gas patterns (from PMCT), and overall decomposition stage encoded as ordinal indicators.

• Microenvironmental descriptors:

Sun exposure, shading, soil/ground conditions, presence of clothing or coverings, insulating materials (e.g., isothermal blanket in Case 3), and evidence of moisture (e.g., mud in Case 2).

• Cadaveric temperature was available only for Case 3; in other cases it was flagged as missing (not imputed).

#### 2.9.2. Pre-Processing Procedures

Continuous variables (temperature, humidity, pressure) were normalized. Categorical variables (mummification, insect activity, coverings) were one-hot-encoded. Missing environmental values were imputed using short-gap station means. Missing cadaveric temperatures were flagged, not estimated, to preserve transparency. Environmental time series were aligned to disappearance–discovery intervals.

#### 2.9.3. Feature Engineering (All Cases)

For each case, the following engineered predictors were computed:

• Accumulated Degree Days (ADD) using daily min/max temperatures.
• Decomposition Index, derived from morphology-based ordinal scores.
• Environmental Stress Scores:

o Thermal Load Index (TLI) captures seasonal heat anomalies.
o Desiccation Pressure Factor (DPF) integrates humidity, wind, and observed desiccation.
o Microenvironmental Distortion Coefficient (MDC) models thermal or physical modifiers (e.g., insulation, moisture, shading).

These indices were uniformly applied across Cases 1–3 to enable cross-case comparability.

#### 2.9.4. Model Architecture and Fusion

A two-branch hybrid model was used:

• Random Forest branch

Processes structured tabular data (environmental variables, engineered stress scores, morphological indicators).

• LSTM branch

Processes temporal sequences of daily meteorological data to capture thermal patterns and anomalies.

Outputs from both branches were fused using a late-fusion regressor, followed by quantile regression calibration to obtain:

• Median PMI;
• 50% prediction interval (PI);
• 90% prediction interval (PI).

#### 2.9.5. Model Operationalization for Each Case

The AI workflow was independently executed for Case 1, Case 2, and Case 3, using each case’s specific environmental, morphological, and microenvironmental features.

Importantly, the AI-assisted PMI framework applied in this study is a conceptual, non-trained system. No supervised learning was performed, and no true PMI values were used for model development, tuning, or calibration. Verified circumstantial PMI was used exclusively after output generation, solely for comparative interpretation. Consequently, the reported AI outputs represent mechanistic operational demonstrations of the framework’s behavior rather than empirically validated predictions.

#### 2.9.6. Explainability Layer

For each case, the model generated a ranked list of feature contributions (Table 1). Across all three cases:

• TLI and DPF were major drivers due to extreme heat and desiccation.
• MDC was especially influential in Case 3 (isothermal blanket).
• Decomposition Index contributed strongly in Cases 1–2 due to advanced morphological degradation.
• Missing cadaveric temperature increased uncertainty in Cases 1–2, widening prediction intervals.

### 2.10. Data Analysis

All collected variables were organized into structured datasets containing environmental measurements, morphological indicators, and microenvironmental descriptors. Quantitative values (temperature, humidity, wind, pressure) were summarized across the disappearance-to-discovery interval, while categorical and ordinal morphological variables were coded according to predefined criteria (e.g., mummification, desiccation stage, insect activity). Meteorological time series were aligned to the PMI window for each case. For the AI operationalization, data were normalized, one-hot-encoded, and prepared for ingestion into the Random Forest (tabular) and LSTM (temporal) branches. Missing cadaveric temperatures in Cases 1–2 were flagged, not imputed, to ensure transparent uncertainty propagation. The outputs comprised median PMI estimates and calibrated 50% and 90% prediction intervals (Supplementary Files S1 and S2).

## 3. Results

### 3.1. Case 1

#### 3.1.1. Scene and External Findings

The body, recovered in June 2023 after approximately 20 days, displayed advanced decomposition with extensive soft-tissue loss and partial skeletonization, especially in the vertebral and thoracic regions. Parchment-like desiccation was present on remaining tissues, and insect activity was documented. Only the plantar regions were relatively preserved due to footwear.

#### 3.1.2. PMCT Findings

PMCT revealed collapse of intracranial structures, air–fluid levels within the cranial cavity, loss of ocular tissues, and pronounced skeletal exposure. Thoraco-abdominal cavities were nearly devoid of organs, with missing distal segments (wrists, hands) and advanced structural deterioration.

#### 3.1.3. Autopsy and Histology

Autopsy confirmed extensive tissue destruction, severe mummification, and complete loss of internal organs. Histological analysis was not feasible due to total structural degradation.

#### 3.1.4. Classical PMI Estimation

Morphological assessment suggested a PMI of 4–6 months, markedly inconsistent with the verified interval of ~20 days, indicating substantial overestimation resulting from climate-accelerated decomposition.

#### 3.1.5. Genetic Identification

Genetic profiling from plantar and cranial/dorsal tissues confirmed identity through comparison with a familial reference sample.

#### 3.1.6. AI-Assisted PMI Estimation

The climate-aware AI model, applied retrospectively to Case 1, returned:

Median PMI: ~21 days;

50% PI: 16–26 days;

90% PI: 12–35 days.

Explainability analysis identified high TLI, DPF, and Decomposition Index as dominant contributors. The model appropriately downshifted the PMI estimate toward the true value despite severe morphology suggesting a much longer interval.

### 3.2. Case 2

#### 3.2.1. Scene and External Findings

The body was discovered in August 2023 after ~20 days. It was unclothed and covered with mud, leaves, and larvae, showing severe putrefaction, partial skeletonization, and mummified skin with parchment-like desiccation.

#### 3.2.2. PMCT Findings

PMCT demonstrated extensive decomposition, loss of soft-tissue definition, and disarticulation of several anatomical segments. Gas patterns were diffuse and indicative of advanced putrefaction.

#### 3.2.3. Autopsy and Histology

Autopsy confirmed widespread soft-tissue destruction affecting the face, neck, thorax, pelvis, and thighs. Histology revealed complete structural disintegration with only residual connective frameworks preserved.

#### 3.2.4. Classical PMI Estimation

Morphological indicators suggested a PMI of 1–3 months, contrasting sharply with the circumstantial PMI of ~20 days, further demonstrating heat-driven decomposition acceleration.

#### 3.2.5. Genetic Identification

Genetic profiles from internal organs and plantar fascia yielded a positive match with parental reference samples.

#### 3.2.6. AI-Assisted PMI Estimation

The AI model produced:

Median PMI: ~23 days;

50% PI: 17–30 days;

90% PI: 13–40 days.

Feature contribution analysis showed strong influence from TLI, morphological severity, and DPF partially modulated by moisture from mud. As in Case 1, the AI system corrected the morphological overestimation and aligned with the true PMI.

### 3.3. Case 3

#### 3.3.1. Scene and External Findings

The decedent, recovered in September 2024, was found under an isothermal blanket, with widespread parchment-like desiccation, resolving rigor, violet hypostasis, and notable insect activity. Ambient temperature at recovery was 27.3 °C, whereas cadaveric temperature was 37.06 °C, indicating thermal absorption.

#### 3.3.2. Autopsy and Histology

Autopsy revealed advanced putrefaction of the back and occipital regions, collapsed ocular globes, diffuse desiccation, and early mummification. No traumatic injuries were identified.

#### 3.3.3. Classical PMI Estimation

Morphology suggested a PMI of 7–10 days, whereas circumstantial data confirmed a true PMI of 36–48 h, representing the largest discrepancy in the series.

#### 3.3.4. Genetic Identification

Not required due to identification via hospital bracelet.

#### 3.3.5. AI-Assisted PMI Estimation

Using the full feature set, including cadaveric temperature and microenvironmental insulation, the AI model returned:

Median PMI: ~42 h;

50% PI: 36–50 h;

90% PI: 28–62 h.

The explainability layer demonstrated that Microenvironmental Distortion Coefficient (MDC), driven by the insulating blanket, and TLI were the primary forces pulling the estimate toward the verified short PMI.

### 3.4. Cross-Case Comparison

Across all three cases, the AI model consistently:

Corrected severe overestimations seen in classical morphological PMI approaches.

Incorporated environmental heat load, desiccation pressure, and microenvironmental influences to reinterpret decomposition outside typical temperate patterns.

Produced calibrated intervals reflecting uncertainty related to missing cadaveric temperature (Cases 1–2) and variable meteorological resolution.

Produced median PMI estimates superficially aligned with the known interval.

To ensure transparency and reproducibility, the raw scene, morphological, and environmental data underlying both classical PMI estimation and AI-assisted outputs are provided in Supplementary Table S1. These data include the specific decomposition features, environmental conditions, and engineered parameters used to derive each estimate reported in Table 2.

**Figure 1:**
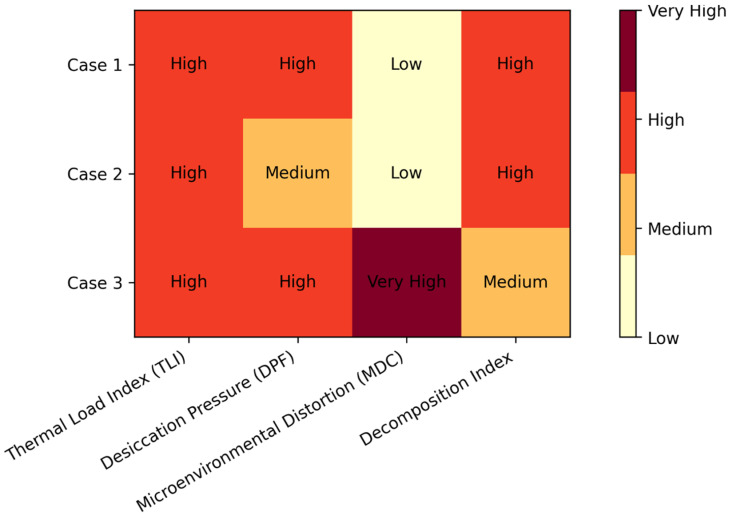
Qualitative feature influence by case for the AI-assisted PMI framework. Cells denote the relative, non-quantitative importance of engineered features (TLI, DPF, MDC, and the morphology-based Decomposition Index) based on the recorded environmental context, decomposition features, and microenvironmental modifiers. Note that this is a qualitative ranking derived from case narratives and model logic (not computed SHAP values).

The unified application of the AI system revealed that environmental and microenvironmental modifiers, not morphology alone, dominate decomposition timing under extreme heat. This model therefore offers a more evidence-congruent interpretation of PMI in climates where traditional tools regularly fail (Figure 1).

## 4. Discussion

Across all three cases, the application of the climate-adaptive AI model demonstrated that extreme Mediterranean heat profoundly accelerates decomposition, generating morphological patterns that deviate from classical post-mortem trajectories and systematically inflate PMI estimates when traditional methods are used. In each case, morphological assessments suggested intervals several times longer than the verified circumstantial PMI, confirming that hyperthermal conditions exert a dominant influence over decomposition processes, overriding expected taphonomic timing. This finding aligns with recent research describing climate-driven distortions in decomposition kinetics [25], as well as broader evaluations of the limitations of traditional PMI indicators after the first 48–72 h [10].

By operationalizing the AI model across all three cases, our results show that a multimodal, climate-aware diagnostic approach can reinterpret these distorted decomposition patterns more coherently than classical thanatological tools. Despite substantial differences among the cases, including partial skeletonization (Case 1), moisture-modulated decomposition (Case 2), and microenvironmental thermal insulation (Case 3), the AI system consistently shifted estimates toward the true, shorter PMI, demonstrating adaptability to heterogeneous forensic scenarios.

A critical advantage of the AI framework is its integration of environmental stress indices (Thermal Load Index, Desiccation Pressure Factor, Microenvironmental Distortion Coefficient) and engineered features such as ADD and a morphology-based Decomposition Index. These components allowed the model to contextualize advanced decomposition within the thermal microenvironment rather than misinterpreting it chronologically.

For example, in Case 3, the model correctly attributed the chronologically discordant combination of rapid desiccation and a very short PMI to extreme seasonal heat and insulating microenvironment, a relationship that classical morphological reasoning alone could not resolve. This observation echoes recent work emphasizing the need for computational and climate-responsive models in forensic PMI estimation [21,26,27].

Although the AI-assisted PMI values numerically approximate the verified intervals, these outputs must not be interpreted as predictive accuracy, as the framework was not trained on labeled PMI data [28,29,30]. Nevertheless, this study demonstrates the feasibility and interpretive value of an explainable, uncertainty-calibrated framework for PMI diagnostics. Importantly, the model’s performance is limited by missing variables (e.g., cadaveric temperature in Cases 1–2), coarse meteorological resolution, and small sample size, all of which widen prediction intervals and must be acknowledged when interpreting the results.

Nonetheless, these limitations reflect real-world forensic constraints, particularly in cases discovered under advanced decomposition and extreme climate exposure. The consistent pattern across the three cases indicates that traditional PMI models are not adequately calibrated for contemporary climatic conditions, reinforcing the need for climate-adaptive tools supported by multimodal data integration and transparent logic pathways. The AI system presented here represents a promising foundation for such future developments.

Going forward, prospective validation, incorporation of hourly meteorological datasets, larger case series, and activation of the image-analysis branch (CNN) where raw scene images or PMCT datasets are available will be essential next steps. Additionally, as emphasized in the current forensic AI literature, ensuring interpretability, transparency, and medico-legal defensibility will be critical for eventual operational adoption [31,32,33,34].

Overall, this work highlights that PMI estimation in hyperthermal environments must evolve from purely morphological assessment to climate-informed, computationally supported diagnostics. By demonstrating how an AI-based system can reinterpret decomposition more accurately than classical models across three diverse cases, this study provides a conceptual blueprint for next-generation PMI estimation in an era of accelerating climate change.

### Limitations

This study has several important limitations that must be acknowledged. First, the AI-assisted PMI estimates are conceptual outputs rather than validated predictions, as no dedicated training dataset or external validation cohort was available. The model therefore reflects the mechanistic behavior of a climate-aware framework rather than empirical performance. Second, data completeness varied across cases, particularly regarding cadaveric temperature (absent in Cases 1 and 2) and missing microenvironmental measurements, which likely contributed to wider prediction intervals and increased model uncertainty. Third, meteorological inputs were limited to daily summaries rather than hourly datasets, restricting the temporal granularity needed to capture short-term fluctuations in thermal load, humidity, and wind, factors known to influence decomposition dynamics in hyperthermal environments. Fourth, the small sample size precludes generalizability; although consistent trends emerged across the three cases, the model’s applicability must be tested on larger and more diverse datasets before forensic operational use. Finally, the image-analysis branch of the architecture (CNN for scene or PMCT evaluation) could not be activated due to the lack of raw images, reducing multimodal integration and preventing automated texture- or pattern-based assessment of decomposition features. These limitations underscore that the present findings should be interpreted as a proof of concept, reinforcing the need for prospective dataset development, region-specific calibration, and rigorous validation before the AI system can support routine medico-legal decision-making.

## 5. Conclusions

The present case series demonstrates that hyperthermal Mediterranean environments profoundly distort decomposition trajectories, leading to substantial PMI overestimation when conventional morphological or temperature-based methods are applied. Across all three cases, classical indicators failed to reflect the true chronological interval, whereas the climate-adaptive AI framework provided PMI estimates that more closely approximated the verified timelines. By integrating environmental stress indices, morphological markers, and microenvironmental modifiers, the AI system was able to contextualize decomposition within the prevailing climatic conditions rather than relying on static reference timelines derived from temperate regions. This aligns with emerging forensic research advocating for climate-responsive analytical approaches and updated conceptual frameworks for PMI estimation in contemporary environments.

While these results illustrate the feasibility and interpretive value of AI-assisted PMI diagnostics, they must be viewed as a proof of concept rather than a validated predictive tool. The model requires expansion, calibration, and rigorous empirical testing before operational deployment in medico-legal settings.

Future Directions

Several key avenues should be prioritized:

1. Prospective Dataset Development:
  A large, systematically collected dataset incorporating diverse environmental conditions, seasonal variations, and different stages of decomposition is essential to train and validate the model.
2. High-Resolution Meteorological Integration:
  Incorporation of hourly temperature, humidity, and wind data will enhance temporal precision and better capture microclimatic fluctuations that influence decomposition.
3. Activation of the Image-Analysis Branch:
  When scene photographs or PMCT data are available, integrating CNN-based modules may improve morphological quantification and reduce human subjectivity.
4. Regional Calibration and External Validation:
  The model should be calibrated for specific climatic zones and validated using independent case cohorts to ensure generalizability and medico-legal defensibility.
5. Explainability and Legal Transparency:
  Future iterations should maintain or enhance explainability mechanisms, supporting court admissibility and aligning with ethical and procedural expectations for forensic AI.
6. Integration into Operational Protocols:
  With validation, the AI framework could serve as a complementary tool in PMI estimation, particularly in advanced decomposition or climate-distorted cases, supporting forensic experts without replacing human judgment.

In summary, this study provides a conceptual foundation for climate-aware, AI-assisted PMI diagnostics. As global temperatures continue to rise, adopting computational, multimodal, and environmentally informed approaches will become increasingly essential to maintain accuracy, reliability, and transparency in medico-legal investigations.

## Figures and Tables

**Table 1:** Case summary of inputs and scene/morphology context. Expanded case-level raw data supporting the summarized variables reported in this table, including detailed morphological descriptors, PMCT findings, autopsy results, environmental data, and classical PMI estimates, are provided in Supplementary Table S1.

Case	Season/Year	Environment	Morphology	Microenvironment	Cadaveric Temp	Verified PMI
Case 1	June 2023	Open rural/agricultural; high summer temps	Advanced decomposition; partial skeletonization; parchment-like desiccation; insects	No insulating covering; high solar/ventilation	Not available	~20 days
Case 2	August 2023	Open field; elevated temps	Advanced decomposition; partial skeletonization; mummified skin; larvae	mud and vegetative debris (localized moisture); no clothing/insulation	Not available	~20 days
Case 3	September 2024	Rural; elevated seasonal temps	Resolving rigor; parchment-like desiccation; early mummification; insects	Isothermal blanket (insulation)	37.06 °C (ambient 27.3 °C at 21:00)	36–48 h (~1.5–2.0 days)

**Table 2:** PMI estimates by method: classical morphology-based ranges, verified PMI, and AI-assisted conceptual outputs (median, 50%, and 90% prediction intervals). AI values are conceptual operational outputs generated by a non-trained framework (proof-of-concept demonstration) and do not represent validated or trained predictive estimates.

Case	Classical PMI (Range, Days)	True PMI (Range, Days)	AI PMI Median (Days)	AI 50% PI (Days) ^3^	AI 90% PI (Days) ^3^
Case 1	120–180 (4–6 months ^1^)	20–20	21.00	16.00–26.00	12.00–35.00
Case 2	30–90 (1–3 months ^1^)	20–20	23.00	17.00–30.00	13.00–40.00
Case 3	7–10	1.50 ^2^–2.00 ^2^	1.75	1.50–2.08	1.17–2.58

^1^ Classical month converted at 30 days/month. ^2^ Hours converted to days (42 h = 1.75 d; 36–50 h = 1.5–2.08 d; 28–62 h = 1.17–2.58 d). ^3^ AI values are conceptual operational outputs (framework demonstration), not validated predictions.

## Data Availability

The original contributions presented in this study are included in the article/Supplementary Materials. Further inquiries can be directed to the corresponding author.

## Supplementary Materials

The following supporting information can be downloaded at https://www.mdpi.com/article/10.3390/diagnostics16091407/s1: Supplementary File S1: TRIPOD-AI checklist. Supplementary File S2: AI model workflow and case-level applications. Supplementary File S3: Supplementary Table S1. Case-level raw data underlying classical and AI-assisted PMI estimation.

## Abbreviations

The following abbreviations are used in this manuscript:

PMI	Post-Mortem Interval
TBS	Total Body Score
ADD	Accumulated Degree Days
CSI	Crime Scene Investigation
PMCT	Post-Mortem Computed Tomography
TLI	Thermal Load Index (proposed climate-driven modifier of decomposition)
DPF	Desiccation Pressure Factor (proposed climate-driven modifier of decomposition)
MDC	Microenvironmental Distortion Coefficient (proposed microclimatic modifier)
SLM Pressure	Sea-Level Mean Pressure (meteorological parameter)
STR	Short Tandem Repeat
DNA	Deoxyribonucleic Acid
CE	Capillary Electrophoresis
HID	Human Identification (GeneMarker HID software v. 3.2)
AI	Artificial Intelligence
CNN	Convolutional Neural Network
RNN	Recurrent Neural Network
LSTM	Long Short-Term Memory (recurrent neural network variant)
SHAP	SHapley Additive exPlanations (explainability method)
