# Supplementary material for: Estimating the Post-Mortem Interval Under Extreme Heat Environments: A Climate-Adaptive Case Series Based on Artificial Intelligence-Supported Diagnostics
Source: Diagnostics (Basel). 2026 May 6;16(9):1407. doi: 10.3390/diagnostics16091407 (PMC13163471; doi:10.3390/diagnostics16091407)
Supplement: Supplementary file 1 [file diagnostics-16-01407-s001.zip › S1_TRIPOD‐AI Checklist.pdf]

Supplementary file

Supplementary File — TRIPOD-AI Checklist (Completed for This Study)

(Transparent Reporting of a Multivariable Prediction Model for Individual Prognosis or Diagnosis: AI Extension)

Study type: Multimodal AI-assisted estimation of post-mortem interval (PMI) in three forensic hyperthermal cases.

Section 1 — Title & Abstract

1. Title

✓ Identifies the study as evaluating a multivariable prediction model and its application to PMI diagnostics in forensic settings.

(TRIPOD-AI requirement: clearly state model type, purpose, and domain) [bmj.com]

2. Abstract

✓ Structured format with Background/Objectives, Methods, Results, Conclusions.

✓ Mentions the model, predictors, outcomes, and uncertainty intervals.

✓ Notes conceptual nature of the model outputs.

Section 2 — Introduction

3. Background & Objectives

✓ Clinical/forensic context described: PMI estimation challenges in hyperthermal environments.

✓ Rationale for developing/applying an AI-assisted diagnostic model provided.

✓ Objectives clearly stated: (i) document climate-driven PMI misestimation; (ii) demonstrate an AI framework for climate-adaptive PMI diagnostics.

Section 3 — Methods

4. Data Sources

✓ Retrospective forensic case-series (2018–2025).

✓ Sources described: CSI, PMCT, autopsy, histology, genetics, meteorology.

✓ Representativeness discussed (Mediterranean summer hyperthermal conditions).

5. Participant Description

✓ Three forensic cases, with details of decomposition stage and environmental exposure.

✓ Eligibility criteria: summer recovery, advanced decomposition, PMI–morphology discrepancy.

6. Outcome Definition

✓ Target outcome = Post-mortem interval (PMI), defined using verified circumstantial last-seen-alive data.

✓ Time horizon explicitly stated for each case.

7. Predictors

✓ Environmental variables

✓ Morphological descriptors

✓ Microenvironmental modifiers

✓ Engineered features (ADD, Decomposition Index, TLI/DPF/MDC)

8. Missing Data Handling

✓ Missing cadaveric temperatures flagged, not imputed.

✓ Meteorological gaps imputed using short-gap station means.

✓ Documented uniformly across cases.

9. Sample Size

✓ Three cases explained as extreme-scenario demonstrators; model outputs labeled conceptual.

✓ Acknowledgment of small-sample constraints included (recommended by TRIPOD-AI).

10. Model Specification

✓ Hybrid architecture described:

- Random Forest (structured features)

- LSTM (temporal meteorology)

- Late fusion

- Quantile regression calibration

11. Model Training / Validation

✓ Stated clearly: *No external training dataset available; outputs are operational demonstrations rather than validated predictions.*

✓ No cross-validation performed due to lack of training data (transparent disclosure required by TRIPOD-AI).

12. Model Explainability

✓ Explainability approach described:

- Feature-importance ranking

- High influence of TLI, DPF, MDC, and decomposition indices

13. Performance Measures

✓ Median PMI, 50% PI, 90% PI reported.

✓ Emphasis on uncertainty rather than numeric "accuracy" (per conceptual demonstration).

✓ Performance not overstated.

Section 4 — Results

14. Participant Flow and Characteristics

✓ All three cases described in detail (scene, morphology, PMCT, autopsy, genetics).

15. Model Performance

✓ Conceptual outputs provided for all three cases:

- Case 1: ~21 days (PI discussed)

- Case 2: ~23 days

- Case 3: ~42 hours

✓ Prediction intervals included.

✓ Drivers identified (explainability).

16. Model Behavior & Interpretability

✓ Key contributing features outlined for each case.

✓ Explanation of how TLI and MDC shift predictions toward true PMI.

Section 5 — Discussion

17. Limitations

✓ Transparency about conceptual nature of outputs.

✓ Acknowledges lack of training dataset, coarse meteorology, missing cadaveric temperatures.

18. Future Work

✓ Need for prospective dataset creation, regional calibration, and validation.

✓ Need for activation of optional image branch (CNN) when image data available.

Section 6 — Other Information

19. Ethical Compliance

✓ Ethical approval described; secondary judicial data used.

20. Availability of Data, Code, and Materials

✓ Model architecture, workflow diagrams, and engineered-feature definitions included in Supplementary Material.

✓ Code is conceptual; pseudo-code available upon request.

21. Funding / Conflicts

✓ Already provided in the main manuscript.

Authors' Declaration of TRIPOD-AI Compliance

The authors affirm that this study follows the TRIPOD-AI reporting structure to the extent applicable for a conceptual, non-trained, method-demonstration model, and all deviations (due to absence of a training dataset or validation cohort) are transparently declared.

(Framework requirements supported by the TRIPOD-AI statement and expanded checklist.)
