# Supplementary material for: Estimating the Post-Mortem Interval Under Extreme Heat Environments: A Climate-Adaptive Case Series Based on Artificial Intelligence-Supported Diagnostics
Source: Diagnostics (Basel). 2026 May 6;16(9):1407. doi: 10.3390/diagnostics16091407 (PMC13163471; doi:10.3390/diagnostics16091407)
Supplement: Supplementary file 1 [file diagnostics-16-01407-s001.zip › S2_AI Model Workflow & Case Applications.pdf]

SUPPLEMENTARY FILE – Operationalization of the AI-Assisted PMI Framework Across All Cases

This Supplementary File details the operational procedure used to apply the climate-adaptive AI model to Case 1, Case 2, and Case 3.

The pipeline follows the same architecture described previously: a multimodal, climate-aware PMI estimation system using environmental, decomposition-morphological, and microenvironmental variables processed through a hybrid Random Forest – LSTM model with quantile-regression calibration.

Important clarification on model status

The AI framework presented herein is not trained on true PMI labels and does not rely on supervised learning. Outputs are generated through predefined feature transformations, climate-stress indices, and rule-constrained fusion logic. Verified PMI is used only for post-hoc comparison and never as an input.

1. Overview of the AI Framework

The model integrates three data streams:

1. Environmental & meteorological variables
2. Cadaveric & decomposition morphology
3. Microenvironmental modifiers (e.g., insulation, ground surface, shading)

The system includes:

- Pre-processing: normalization, one-hot encoding, missing-data flags.
- Feature engineering:
    - Accumulated Degree Days (ADD) variants
    - Decomposition Index (ordinal morphological features)
    - Environmental Stress Scores:
        - Thermal Load Index (TLI)
        - Desiccation Pressure Factor (DPF)
        - Microenvironmental Distortion Coefficient (MDC)
- Model architecture:
    - Random Forest for structured tabular features
    - LSTM for temporal (daily meteorology) sequences
    - Late fusion + quantile calibration
- Outputs:

- Median PMI
- 50% and 90% prediction intervals
- Explainability: feature importance summary

This pipeline comes directly from your original Supplementary text for Case 3.

2. Data Used in All Cases

All three cases provided:

- Daily meteorological data (mean/min/max temperature, humidity, pressure, wind)
- Morphological indicators (mummification, desiccation, putrefaction, skeletal exposure)
- Insect presence/absence
- Environmental context (sun exposure, terrain, coverings, vegetation)
- Verified circumstantial PMI

Case 3 additionally provided cadaveric temperature and a strongly distorting isothermal blanket.

These elements are fully documented in the manuscript's case descriptions.

3. Case-Specific AI Operationalization

Below, each case is described using the same framework:

Inputs → Pre-processing → Feature engineering → Model fusion → Outputs → Explainability.

3.1. Case 1 – Advanced Skeletonization Under Extreme Heat (20-day true PMI)

3.1.1. Input Features

- Environmental: high summer temperatures (Tmax ≈ 31 °C; elevated seasonal averages).
- Cadaveric/morphological:
    - Extensive soft-tissue loss
    - Partial skeletonization
    - Parchment-like desiccation
    - Insect colonization
- Microenvironment:
    - Open rural/agricultural terrain
    - No insulating coverings
    - High ventilation and solar exposure

(From manuscript's case description.)

3.1.2. Pre-Processing

- Normalization of continuous variables (temperature series).
- Encoding of categorical features (skeletonization, desiccation, fauna).
- Missing cadaveric temperature flagged (not imputed).
- Meteorological short gaps filled with station means.

3.1.3. Feature Engineering

- ADD: computed from station temperature over disappearance–discovery interval.
- Decomposition Index: high (severe morphology, early skeletonization).

  Decomposition Index — Ordinal Feature Engineering Matrix

  Purpose:

  To encode multiple morphological descriptors into a unified ordinal index that summarizes the severity and stage of decomposition for use in the RF branch of the conceptual AI model.

  Scores 0–4 reflect increasing decomposition severity and tissue loss.

**Category**	**Score**	**Operational Definition (Aligned with manuscript & cases)**
**0 – Fresh / Early**	**0**	Minimal external change; no desiccation; rigor/livor may be present; no insect activity.
**1 – Early Decomposition**	**1**	Mild discoloration, initial bloating, limited epidermal slippage; early insect colonization; no mummification.
**2 – Advanced Decomposition (non-desiccating)**	**2**	Widespread putrefaction, strong odor, gas formation, tissue liquefaction, sloughing; organs still structurally identifiable; no parchmenting or mummification. Seen partially in **Case 3** (back/occipital putrefaction).
**3 – Desiccation / Early Mummification**	**3**	Parchment-like skin, drying of exposed surfaces, partial preservation of deeper tissues; early mummification; limited skeletal exposure; insect activity present. Seen in **Case 3** (parchmenting + early mummification).
**4 – Advanced Mummification / Partial Skeletonization**	**4**	Deep desiccation, stiff parchment-like remains, extensive soft-tissue loss, partial skeletonization of limbs or torso, loss of internal organs, advanced insect activity. Corresponds to **Cases 1 and 2** (severe destruction + partial skeletonization + complete organ loss).

- Stress Scores:
  - TLI: high due to heat anomalies relative to 2018–2013–2008 datasets.
  - DPF: high (desiccation + wind).

- MDC: low (no insulation).

3.1.4. Model Fusion & Calibration

- RF processes morphology + stress + static environment features.
- LSTM processes temperature sequence.
- Fused output is calibrated via quantile regression.

3.1.5. Output (Conceptual)

- Median PMI: ~21 days
- 50% PI: 16–26 days
- 90% PI: 12–35 days

3.1.6. Explainability

Dominant predictors:

- TLI (seasonal heat anomaly)
- DPF (rapid desiccation)
- High Decomposition Index

These pull the model toward a *shorter* PMI than expected from morphology, aligning with the true ~20 days.

3.2. Case 2 – Mud-Covered, Open-Air Decomposition (20-day true PMI)

3.2.1. Input Features

- Environmental: high summer temperatures similar to Case 1.
- Morphology:
  - Severe soft-tissue destruction
  - Partial skeletonization
  - Mummified skin
  - Larvae and insect activity
- Microenvironment:
  - Mud, leaf debris → localized moisture
  - No clothing or insulating covering
  - Outdoor exposure

3.2.2. Pre-Processing

Same as Case 1. Mud and moisture encoded as microenvironmental descriptors.

3.2.3. Feature Engineering

- ADD: summer interval.
- Decomposition Index: high.
- Stress Scores:
    - TLI: high
    - DPF: mixed—high heat but moderated by mud moisture
    - MDC: low (no insulation)

3.2.4. Model Fusion & Calibration

Same hybrid ensemble as Case 1.

3.2.5. Output (Conceptual)

- Median PMI: ~23 days
- 50% PI: 17–30 days
- 90% PI: 13–40 days

3.2.6. Explainability

Main contributors:

- TLI (regional heat)
- Morphology severity
- Mud-modulated DPF, preventing excessive desiccation

The AI model captures the environmental acceleration and avoids the extreme overestimation produced by classical morphology.

3.3. Case 3 – Rapid Desiccation + Insulation (36–48 h true PMI)

This section expands the original Supplementary material.

3.3.1. Input Features

- Environmental:
    - Ambient 27.3 °C at recovery
    - Seasonal maxima ≈ 32.7 °C (higher than 2019–2014–2009)
- Morphology:
    - Resolving rigor
    - Parchment-like desiccation
    - Early mummification

  - Insect activity
- Microenvironment:
  - Isothermal blanket producing thermal insulation
  - Evening recovery time
  - Ground exposure

3.3.2. Pre-Processing

As in the original supplement: normalization, encoding, missing-temperature flags not used (cadaveric temp available).

3.3.3. Feature Engineering

- ADD: based on ambient series.
- Decomposition Index: moderate-to-high (discordant with true PMI).
- Stress Scores:
  - TLI: high (seasonal heat).
  - DPF: moderate-high due to dryness + wind.
  - MDC: very high (insulation).

3.3.4. Model Fusion & Calibration

Same architecture as above.

3.3.5. Output (Conceptual)

- Median PMI: ~42 hours
- 50% PI: 36–50 hours
- 90% PI: 28–62 hours

3.3.6. Explainability

Primary drivers:

- Microenvironmental Distortion (MDC): insulation traps heat → rapid surface desiccation.
- Seasonal TLI: elevated 2024 temperatures.

  This shifts the estimate toward the known 36–48 h interval, resolving the paradox of "long-interval morphology" from classical assessment.

4. Cross-Case Comparison

Across Cases 1–3:

- The AI system consistently shifted PMI estimates toward the true, shorter intervals, correcting the overestimation produced by morphology-based methods.

- TLI was the dominant factor in all cases (extreme summer heat).
- DPF modulated differently: high in Cases 1–3, but partially mitigated by moisture in Case 2.
- MDC was the decisive differentiator in Case 3 (insulation).
- Prediction intervals appropriately widened when key data (cadaveric temperature, intact organs) were missing.

This demonstrates the generalizability and interpretability of the AI model across distinct hyperthermal forensic scenarios.

5. Important Note on Interpretation

These AI outputs are conceptual operational demonstrations, not validated predictions.

They illustrate how a climate-aware multimodal model interprets forensic decomposition under extreme environmental distortion. Prospective dataset creation and rigorous validation are required before operational deployment.
