# Supplementary material for: Estimating the Post-Mortem Interval Under Extreme Heat Environments: A Climate-Adaptive Case Series Based on Artificial Intelligence-Supported Diagnostics
Source: Diagnostics (Basel). 2026 May 6;16(9):1407. doi: 10.3390/diagnostics16091407 (PMC13163471; doi:10.3390/diagnostics16091407)
Supplement: Supplementary file 1 [file diagnostics-16-01407-s001.zip › S3_Supplementary Table S1.pdf]

Supplementary Table S1. Case-level raw data underlying classical and AI-assisted PMI estimation.

Case	Verified PMI (circumstantial)	Scene & Recovery Context	Key Morphological Findings (Raw Data)	CT / Autopsy Findings (Raw Data)	Classical PMI Estimate ([1–3])	Environmental & Climatic Context (Raw Data)	AI-Relevant Inputs Derived from Raw Data*
**Case 1**	~20 days (last sighting: 29 May 2023; recovery: 19 June 2023)	Open agricultural field, rural area near Catania (Sicily); outdoor exposure; foreign materials present	Advanced decomposition; extensive soft-tissue loss; partial skeletonization; parchment-like skin desiccation ("tanned leather" effect); larval colonization	CT: cerebral collapse with air–fluid levels; absence of ocular bulbs; severe facial soft-tissue loss; vertebral and thoracic skeletonization; loss of wrists/hands; absent costal cartilages. Autopsy: absence of internal organs	4–6 months	Summer period; progressive increase in minimum, mean, and maximum temperatures compared with 5-, 10-, and 15-year historical data	ADD: high; Decomposition Index: high; TLI: high; DPF: high (desiccation + heat); MDC: low (no insulation); Cadaveric temperature: not available
**Case 2**	~20 days (last sighting: 31 July 2023; recovery: 17 August 2023)	Open agricultural field near Catania; body unclothed; covered by mud, leaves, larvae	Severe putrefaction; partial skeletonization; extensive soft-tissue loss; mummified, parchment-like skin (yellow-brown coloration);	CT: advanced putrefaction with loss of morphological definition; disarticulation of multiple segments. Autopsy: near-complete loss of soft tissues in	1–3 months	Summer heatwave period; temperature records higher than historical averages for same timeframe	ADD: high; Decomposition Index: high; TLI: high; DPF: moderate (heat counterbalanced by mud-related moisture); MDC: low (no insulation); Cadaveric

			abundant necrophagous fauna	face, neck, thorax, pelvis; connective tissue remnants only			temperature: not available
**Case 3**	36–48 hours (last sighting: 31 August 2024; recovery: 2 September 2024)	Open agricultural field near Acireale; body covered with isothermal blanket	Resolving rigor mortis; violet hypostasis fixed; diffuse parchment-like desiccation; early mummification; insect colonization	Autopsy: putrefaction predominant on back and occipital region; collapse of eyeballs; diffuse "tanned leather" skin; macrofauna colonization	7–10 days	Ambient temperature at discovery: 27.3 °C (21:00); cadaveric temperature: 37.06 °C; marked temperature anomalies versus 5-, 10-, and 15-year historical data	ADD: moderate; Decomposition Index: moderate–high (discordant with PMI); TLI: high; DPF: moderate-high; MDC: very high (thermal insulation from blanket); Cadaveric temperature: available

* AI-relevant inputs were derived deterministically from the reported raw data and used within a conceptual, non-trained framework. No true PMI values were used as inputs at any stage. AI outputs reported in Table 2 represent mechanistic operational demonstrations rather than validated predictions.

References

1. Pittner, S.; Bugelli, V.; Eric Benbow, M.; Ehrenfellner, B.; Zissler, A.; Campobasso, C.P.; Oostra, R.J.; Aalders, M.C.G.; Zehner, R.; Lutz, L.; et al. The Applicability of Forensic Time since Death Estimation Methods for Buried Bodies in Advanced Decomposition Stages. *PLoS One* **2020**, *15*, doi:10.1371/journal.pone.0243395.
2. Madea, B. Methods for Determining Time of Death. *Forensic Sci. Med. Pathol.* **2016**, doi:10.1007/s12024-016-9776-y.
3. Henssge, C.; Madea, B. Estimation of the Time since Death. *Forensic Sci. Int.* **2007**, doi:10.1016/j.forsciint.2006.05.017.
